# Tumor secreted ANGPTL2 facilitates recruitment of neutrophils to the lung to promote lung pre-metastatic niche formation and targeting ANGPTL2 signaling affects metastatic disease

**DOI:** 10.18632/oncotarget.27433

**Published:** 2020-02-04

**Authors:** Manish Charan, Piyush Dravid, Maren Cam, Bhuvana Setty, Ryan D. Roberts, Peter J. Houghton, Hakan Cam

**Affiliations:** ^1^Center for Childhood Cancer and Blood Diseases, Nationwide Children’s Hospital, Columbus, Ohio, USA; ^2^Department of Hematology & Oncology, Nationwide Children’s Hospital, Columbus, Ohio, USA; ^3^Greehey Children’s Cancer Research Institute, Department of Molecular Medicine, University of Texas Health Science Center at San Antonio, San Antonio, Texas, USA; ^4^Department of Pediatrics, The Ohio State University, Columbus, Ohio, USA

**Keywords:** pre-metastatic niche formation, osteosarcoma, ANGPTL2, tumor microenvironment, neutrophils

## Abstract

The pre-metastatic niche (PMN) represents an abnormal microenvironment devoid of cancer cells, but favoring tumor growth. Little is known about the mechanisms that generate the PMN or their effects on host cells within metastasis-prone organs. Here, we investigated by using spontaneous metastatic models whether lung epithelial cells are essential for primary tumor induced neutrophil recruitment in lung and subsequently initiating PMN formation in osteosarcoma. We found that serum levels of ANGPTL2 in osteosarcoma patients are significantly higher compared to those in healthy controls and that ANGPTL2 secretion by tumor cells plays an essential role in osteosarcoma metastasis. We determined that tumor-derived ANGPTL2 stimulates lung epithelial cells, which is essential for primary tumor-induced neutrophil recruitment in lung and subsequent pre-metastatic niche formation. Lastly, we identified that a p63 isoform, ΔNp63, drives high level of ANGPTL2 secretion and pharmaceutical inhibition of ANGPTL2 signaling by a non–RGD-based integrin binding peptide (ATN-161) diminished metastatic load in lungs likely due to reduction of the lung pre-metastatic niche formation.

## INTRODUCTION

Primary tumors selectively and actively modify potential sites of metastasis, even prior to dissemination [1–3]. This “tumor-education” at a distance results when tumor-secreted factors and extracellular vesicles shed by tumor cells elicit changes in distant tissues that facilitate subsequent outgrowth of disseminated cancer cells. For instance, some reports have suggested that lung epithelial cells can respond to tumor-derived signals in ways that trigger pre-metastatic niche formation (reviewed in [1]). However, the mechanisms by which primary tumors affect lung epithelium to promote pre-metastatic niceh formation remain poorly understood.

Type I and type II epithelial cells line the epithelial surface of the lung, serve an immunological barrier function in the respiratory tract, mediate gas exchange, and maintain surface tension to reduce the work of respiration [4]. In addition, lung epithelial cells can detect pathogen- and injury-associated signals, actively generating the first steps of an innate immune response to microbial and other insults [5]. Activation of pattern-recognition receptors (PRRs) by foreign organisms and materials triggers the production of cytokines and chemokines that drive the recruitment of immune cells and subsequent inflammatory processes [6–9]. Neutrophils recruited to an inflammatory metastatic niche often alter their polarity, changing from a tumor-suppressing photype to a tumor-promoting phenotype [10, 11]. While neutrophils have historically been characterized as being important for immune-mediated control of disseminated cells [12], recent reports suggest that tumor-educated neutrophils can suppress both innate and adaptive anti-tumor immune responses [13–15]. For instance, neutrophils have been identified as facilitators of breast cancer metastasis [16] and of lung cancer metastasis after UV-induced inflammation through tumor-secreted exosomal RNAs [17, 18]. Here, we set out to determine how tumor-derived factors might affect the activation of lung epithelial cells in ways that elicit pro-metastatic inflammatory responses and facilitate the formation of the pre-metastatic following recruitment of neutrophils.

ANGPTL2 protein is a secreted glycoprotein that exerts effects through autocrine or paracrine signaling via the integrin alpha5beta1 (α5β1) receptor. Diverse roles of ANGPTL2 in physiology and pathophysiology have been described including cell motility, metastasis, expression of inflammation-related genes, and MMPs [19, 20]. In contrast to induction of inflammation-related genes by ANGPTL2, the roles of cell motility and MMPs in cancer progression are well documented. It is also well established that the induction of inflammation related genes result in activation of neutrophils and as described above a body of evidence suggesting that the recruitment of neutrophils promotes cancer metastasis. Collectively, by using spontaneous metastatic models, we investigated whether tumor secreted ANGPTL2 induces inflammation on lung epithelial cells by activating alpha5beta1 (α5β1) receptor and recruiting neutrophils to the pre-metastatic niche.

## RESULTS

### High level of ANGPTL2 secretion plays a critical role in osteosarcoma metastasis

**Figure 1:**
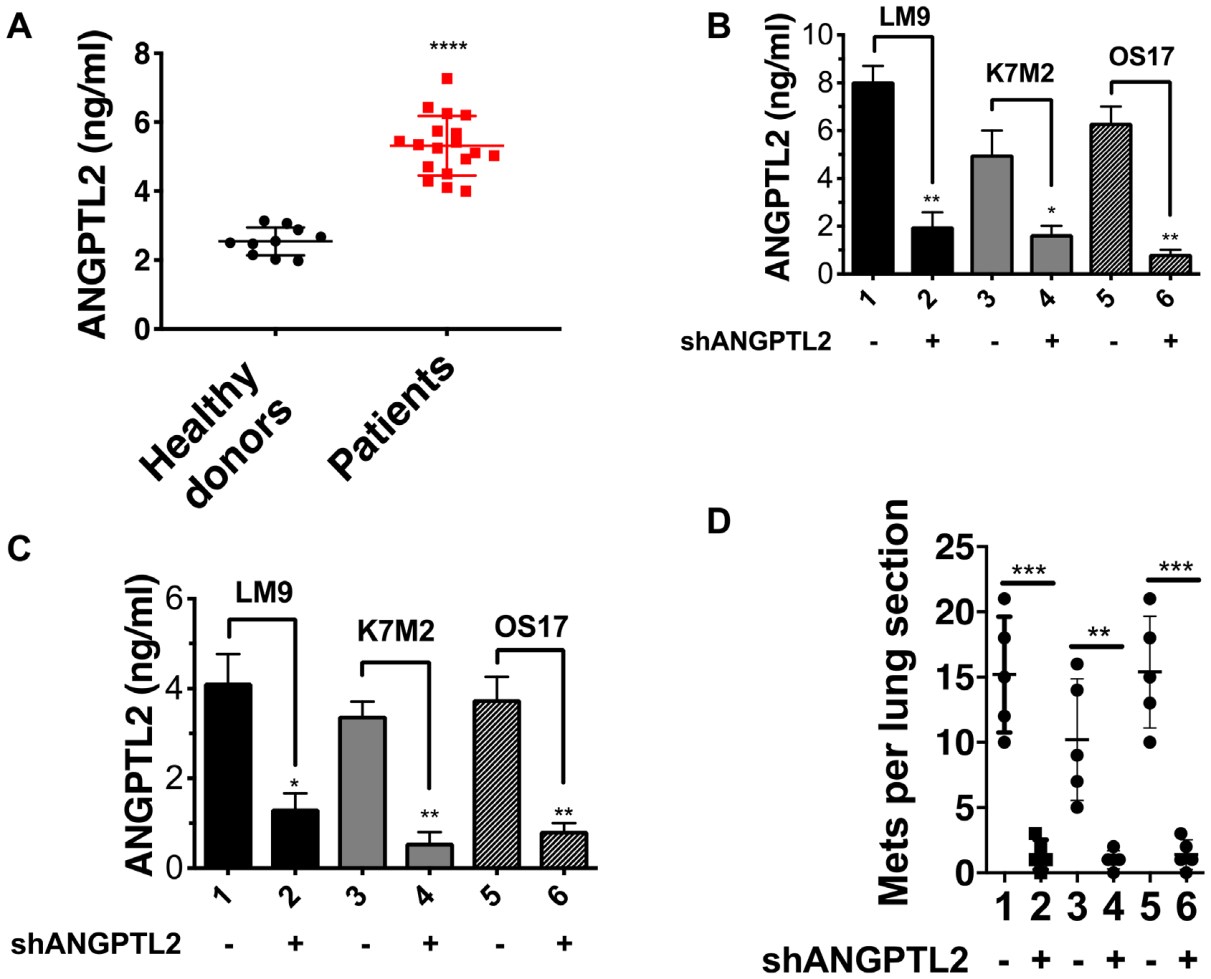
ANGPTL2 plays a crucial role in osteosarcoma metastasis. (**A**) Osteosarcoma patients have high levels of serum ANGPTL2. Serum samples from osteosarcoma patients were obtained at varying stages of osteosarcoma therapy and ANGPTL2 levels were compared to healthy donors. Statistical analyses were performed using GraphPad Prism software. Results are expressed as mean ± SD. ^****^ *P* < 0.0001 versus healthy donors using unpaired Student’s *t*-test with Welch’s correction. (**B**) ANGPTL2 knockdown efficiency in osteosarcoma cells were tested by ELISA as described in Materials and Methods. (**C**) ANGPTL2 levels in serum from tumor-bearing animals (± ANGPTL2 knockdown) was determined after 14 days tumor growth using an ANGPTL2 ELISA kit. (**D**) ANGPTL2 knockdown reduces metastatic lung colonization in the spontaneous metastatic mouse model as described in materials and methods (*n* = 5 for each group). Lanes: 1. LM9-shCtr, 2. LM9-shANGPTL2, 3. K7M2-shCtr 4. K7M2-shANGPTL2, 5-OS17-shCtr, 6-OS17-shANGPTL2. Similar results were obtained by utilizing second shRNA targeting ANGPTL2. Unpaired Student’s *t*-tests, ^*^ *p* < 0.05, ^**^ *p* < 0.01, ^***^ *p* < 0.001.

Given the known actions of ANGPTL2 and the strong propensity of osteosarcoma for metastasis to lung, we asked whether ANGPTL2 might invole in osteosarcoma metastasis. Interestingly, we found that ANGPTL2 levels were strikingly elevated in serum from osteosarcoma patients compared to healthy controls (Figure 1A). We suspected that high level ANGPTL2 secretion might endow these tumors with metastatic potential. We first asked whether high expression of ANGPTL2 in osteosarcoma cell lines correlated with metastatic nodule formation *in vivo*. We found that the OS17 cell line, which secretes high levels of ANGPTL2, formed considerably more lung metastases than OS25 cells, which secrete lower ANGPTL2 (Supplementary Figure 1A–1B).

To test the role of ANGPTL2 in metastasis development, we knocked down ANGPTL2 gene expression in highly metastatic mouse (LM9, K7M2) and human (OS17) osteosarcoma cell lines and verified knockdown efficiency by ELISA (Figure 1B). We then implanted these osteosarcoma cells (with or without ANGPTL2 knockdown) into the tibia of syngeneic (LM9, K7M2) or SCID mice to generate orthotopic tumors and determined serum levels of ANGPTL2 after 2 weeks. Similar to our observations in patients, serum from mice injected with non-manipulated tumor cells (control shRNA) showed high levels of ANGPTL2 (Figure 1C). In contrast, serum ANGPTL2 levels were dramatically lower in mice bearing ANGPTL2-suppressed tumor cells.

In a separate experiment, the same cell lines (with or without ANGPTL2 knockdown) were inoculated into the tibia, let to grow to a pre-determined size, then removed by limb amputation. Eight weeks later lung metastases were evaluated (used animal models are described in Supplementary Figure 2). As shown in Figure 1D, downregulation of ANGPTL2 expression significantly reduced lung metastasis compared to control cells, confirming a functional role for ANGPTL2 in development of spontaneous lung metastasis. In contrast, primary tumor growth rates for LM9, K7M2 and OS17 primary tumors were unaffected by downregulating ANGPTL2 (Supplementary Figure 1C).

### ANGPL2 receptor integrin α5β1 required for the pre-metastatic niche formation

**Figure 2:**
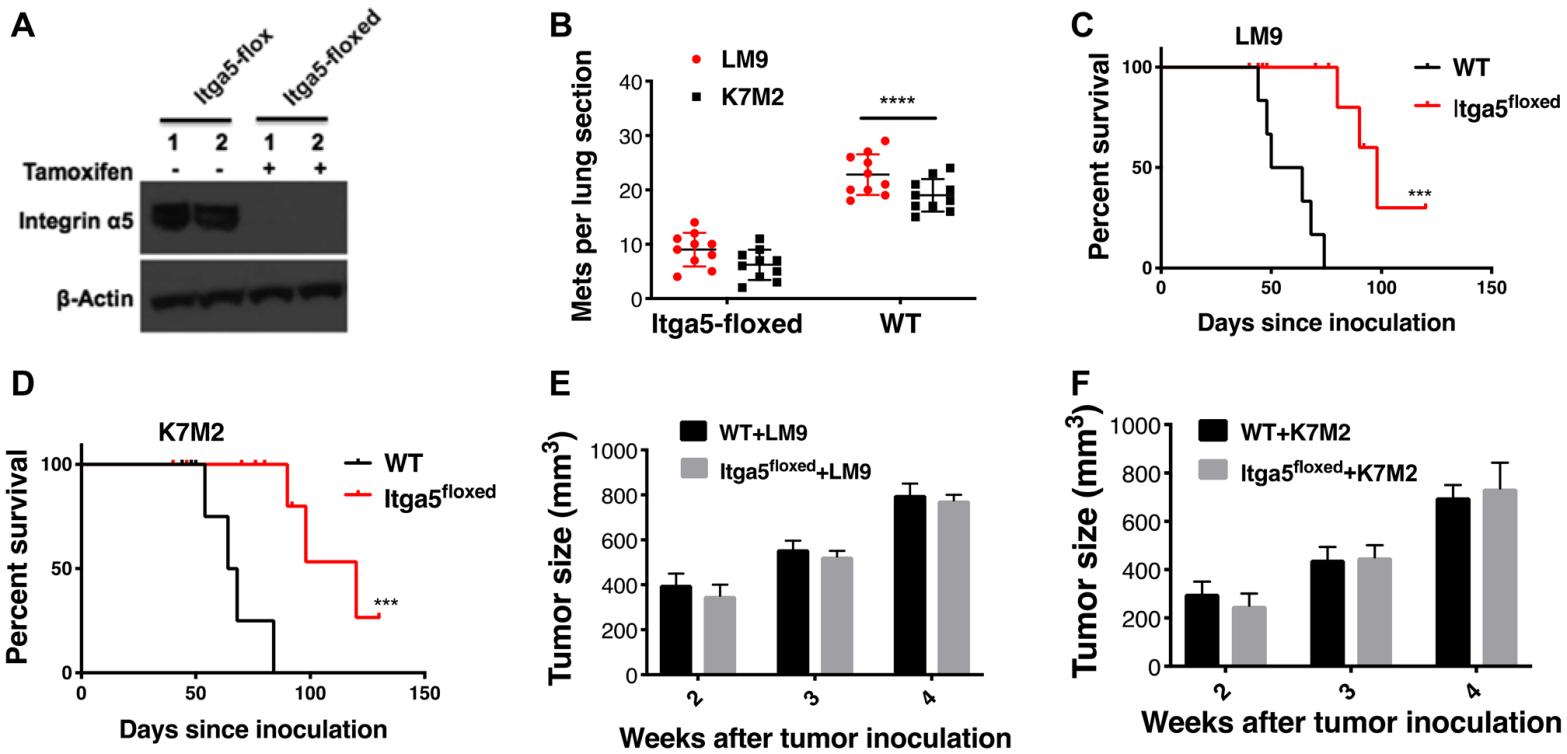
Integrin α5β1 deficiency in alveolar type II (AT-II) diminishes establishment of osteosarcoma lung metastasis. (**A**) To induce tissue specific knockout of integrin 5α in Type II alveolar cells, tamoxifen was administrated. Lung cell suspensions are prepared by intratracheal instillation of dispase and agarose followed by mechanical disaggregation of the lungs. Alveolar type II epithelial cells were purified from these lung cell suspensions through magnetic-based negative selection using a Biotin-antibody, Streptavidin-MicroBeads system. Protein lysate from the purified alveolar type II epithelial cells analyzed by western blot with antibodies as shown. (**B**) Osteosarcoma cells were injected into the tibia of either Itga5-flox (herein, wild-type, WT) or Itga5-floxed mice (*n* = 40, 10 mice for each group). After primary tumors reached around 800 mm^3^ (range from 4 to 5 weeks), primary tumor containing leg was amputated. Animals reaching endpoints were terminated, and lungs were harvested, insufflated, fixed, sectioned, and stained. The number of lung sections with metastatic nodules were compared with ordinary Two-way Anova analysis, Itga5-floxed vs wt-Itga5 (wild-type, WT). ^****^ *P* < 0.0001. (**C–D**). Prolonged survival of mice following deletion of integrin α5β1. ^***^ *P* < 0.001, WT vs Itga5-floxed injected with LM9 and WT vs Itga5-floxed injected with K7M2 by Log-rank (Mantel-Cox) test. (**E–F**) Deletion of integrin α5β1 in the alveolar type II (AT-II) cells does not impact on the primary tumor growth in the tibia. Tumor growth was monitored by caliper measurements for four weeks.

Next, to evaluate the role of ANGPTL2’s receptor integrin α5β1 in the metastatic process, we crossed Itga5 (integrin5α) conditional knockout mice (Taconic) with Sftpc-CreER^T2^ (Jackson Laboratory) to induce time- and tissue-specific knockout of integrin α5 gene in Type II alveolar cells (herein, Itga5-floxed, after tamoxifen administration). Of note, previous research has suggested that alveolar type II cells can promote lung tumor development [21]. Subsequently, we isolated the alveolar type II (AT-II) cells from Itga5-floxed mice and the integrin α5 gene knockout was verified by western blotting (Figure 2A) and immunofluorescence (Supplementary Figure 3). To assess the role of ANGPTL2 receptor integrin α5β1 in the pre-metastatic niche formation, Itga5-floxed mice were inoculated with LM9 or K7M2 osteosarcoma cells into tibia. After these tumors reached a pre-determined size, these limbs were amputated and then observed for signs of lung metastasis. As shown in Figure 2B, we found that Itga5-floxed mice showed significant reduction in lung metastasis compared with integrin α5wild-type (WT) littermates. Furthermore, Itga5-floxed mice demonstrated prolonged survival relative to their WT littermates after tumor removal (Figure 2C–2D). However, these same manipulations did not affect primary tumor growth (Figure 2E–2F). Taken together, these observations indicate that deletion of integrin α5β1 in the alveolar type II (AT-II) cells impairs osteosarcoma lung colonization, but not the growth of primary tumors in the bone.

### Integrin α5β1 receptor deficiency reduces neutrophil recruitment to the lung

**Figure 3:**
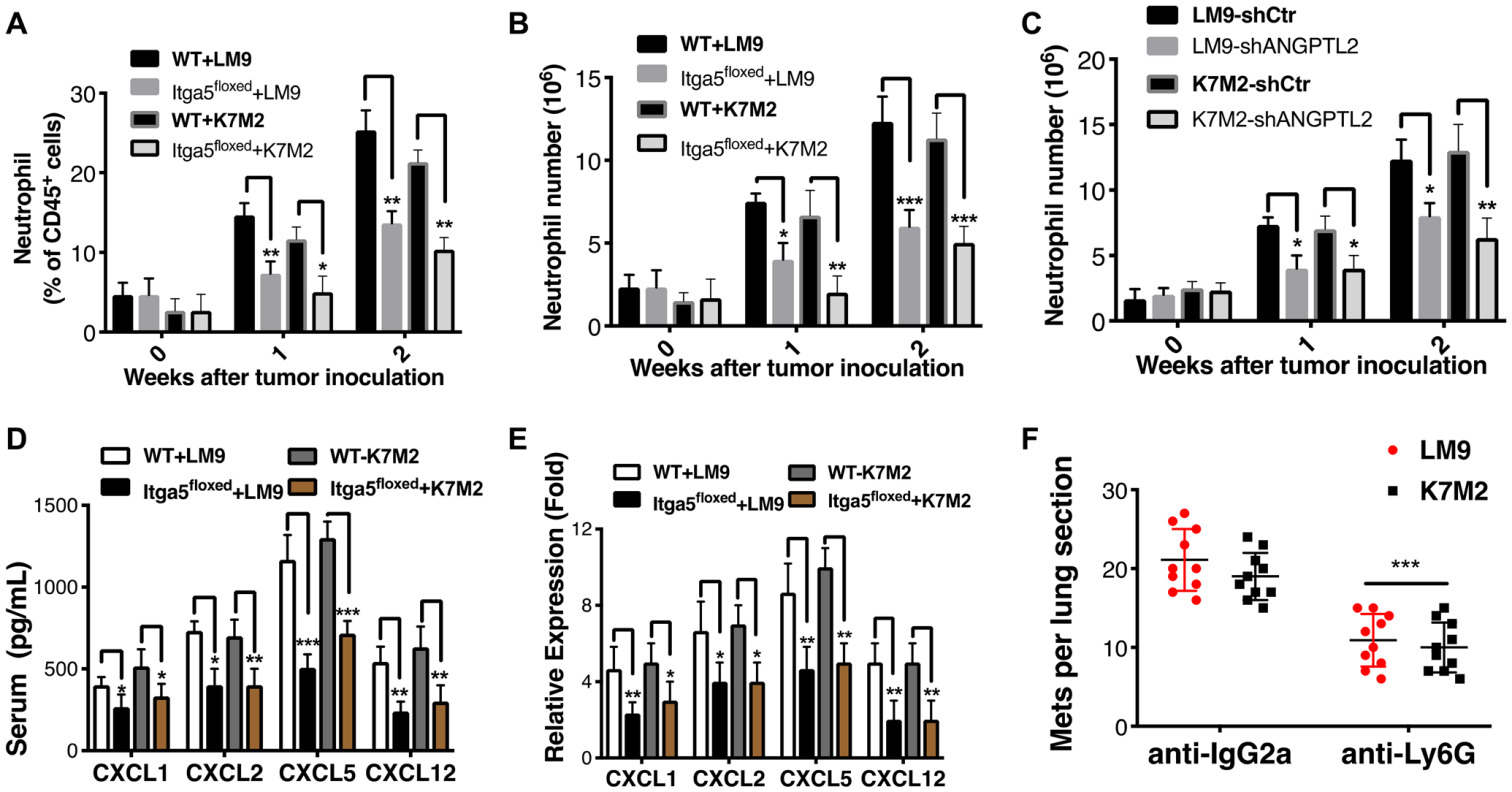
Integrin α5β1 deficiency reduces neutrophil recruitment to the lung. (**A–B**) The proportions (A) and absolute numbers (B) of neutrophils in the lung were detected by flow cytometry in Itga5-floxed mice or WT littermates after with LM9 and K7M2 cells inoculation into tibia (Gating strategy for neutrophils is shown in Supplementary Figure 4). (**C**) Absolute numbers of neutrophils in the lungs were detected by flow cytometry in WT littermates after intratibial inoculation of LM9-shCtr, LM9-shANGPTL2, K7M2-shCtr and K7M2-shANGPTL2 cells at indicated weeks. (**D**) Serum was isolated from tumor bearing animals one week after intratibial injection of tumor cells and analyzed by ELISA according to the manufacturer’s instructions (R&D Systems). Similar results were obtained in BALF (Supplementary Figure 6A–6B) (**E**) CXCL1, CXCL2, CXCL5, and CXCL12 transcripts were significantly lower in Itga5-floxed AT-II cells compared to WT AT-II cells. AT-II cells were isolated from tumor bearing animals after a week of intratibial injection with indicated cells. Subsequently, total RNA was extracted and used for the gene expression analysis of chemokines. Results were normalized to GAPDH. Unpaired Student’s *t*-tests, ^*^ *p* < 0.05, ^**^ *p* < 0.01, ^***^ *p* < 0.001. (**F**) Depletion of neutrophils results in a dramatic reduction of lung metastases. The numbers of lung sections with metastatic nodules were compared with ordinary Two-way Anova analysis analysis, IgG2a vs anti-Ly6G treated. ^***^ *P* < 0.001.

We next examined the downstream signaling pathways that support or initiate pre-metastatic niche following integrin α5β1 receptor activation by ANGPTL2 in AT-II cells. We therefore evaluated whether activation of integrin α5β1 by ANGPTL2 impacted neutrophil recruitment to the lung under physiological conditions. Accordingly, we analyzed lung-infiltrating cellular components in Itga5-floxed and WT littermates at days 1, 7 and 14 after intratibial inoculation with LM9 and K7M2 cells as described in materials and methods. As shown in Figure 3A–3B, CD11b^+^ myeloid cells became much more prevalent in the pre-metastatic lung, with particular enrichment for CD45^+^ CD11b^+^Gr1^+^ neutrophils in WT mice compared to macrophages and dendritic cells (Supplementary Figure 5A–5D). Interestingly, both the relative percentage and absolute number of neutrophils were significantly reduced in the pre-metastatic lungs of Itga5-floxed mice relative to their WT littermates (Figure 3A–3B). These data suggest that neutrophils play a role in pre-metastatic niche formation. Indeed, we still observed an increase of neutrophils in Itga5-floxed mice after inoculation with tumor cells (Figure 3A–3B), suggesting some redundancy in the pathways that mediate neutrophil recruitment to the premetastatic niche. Moreover, we evaluated BV8 expression, a chemokine that is highly expressed in pro-metastatic neutrophils, and described as one of the major components in the metastatic niche [22]. As shown in Supplementary Figure 5E–5F, we found much higher expression of *Bv8* in neutrophils than in macrophages or dendritic cells within tumor-bearing mice, further supporting our finding that neutrophils play a primary role in pre-metastatic niche formation.

### Activation of Integrin α5β1 receptor by ANGPTL2 induces chemokine production that drives neutrophil recruitment

Next, to confirm whether ANGPTL2 indeed activates integrin α5β1 receptor leading to neutrophils recruitment, we injected LM9-shCtr, LM9-shANGPTL2, K7M2-shCtr and K7M2-shANGPTL2 cells into tibia of WT mice. As shown in Figure 3C, the absolute number of neutrophils in the lung were significantly lower in ANGPTL2 depleted tumor cells verifying essential role of tumor secreted ANGPTL2 in neutrophils recruitment. To dissect the underlying mechanisms responsible for impaired neutrophil recruitment in Itga5-floxed mice further, we first analyzed levels of chemokines (CXCL1, CXCL2, CXCL5, and CXCL12) known to stimulate neutrophil chemotaxis. In a separate experiment, serum and bronchoalveolar lavage fluid (BALF) were isolated one week after tibia injections and analyzed with ELISA. As shown in Figure 3D, the secretion of crucial chemokines in the serum was significantly reduced in tumor-bearing Itga5-floxed animals. Moreover, we found that integrin α5β1 deficiency in alveolar type II reduces cytokine expression (Figure 3E). Taken together, our results suggest that activation of lung epithelial α5β1 receptor by ANGPTL2 induces chemokine production that effect neutrophil recruitment in ways that create a tumor-growth-favoring lung microenvironment for disseminated metastatic cancer cells.

In a final experiment, we sought to demonstrate that activated neutrophils were indeed necessary for the pre-metastatic niche formation and establishment of metastasis in osteosarcoma. Neutrophils were depleted by i. p. injection of anti-Ly6G (0.1 mg) monoclonal antibody (Clone 1A8), starting 3 days prior to inoculating and ongoing twice a week for 10 weeks. IgG2a (Clone 2A3) was used as an isotype matched control treatment. LM9 and K7M2 osteosarcoma cells were injected into the tibia of WT mice, 3 days after the initial anti-Ly6G antibody dose (*n* = 40, 10 mice for each group). The leg with primary tumor was amputated at 4–5 weeks after tumor cell inoculation, and animals were euthanized after four weeks. The numbers of metastatic nodules per lung section was compared between groups. As shown in Figure 3F, depletion of neutrophils in tumor bearing animals had significant effects on lung metastasis. Together, these results confirm further that neutrophils are critical for pre-metastatic niche formation in these osteosarcoma models.

### Tumor-secreted ANGPTL2 promotes osteosarcoma extravasation

**Figure 4:**
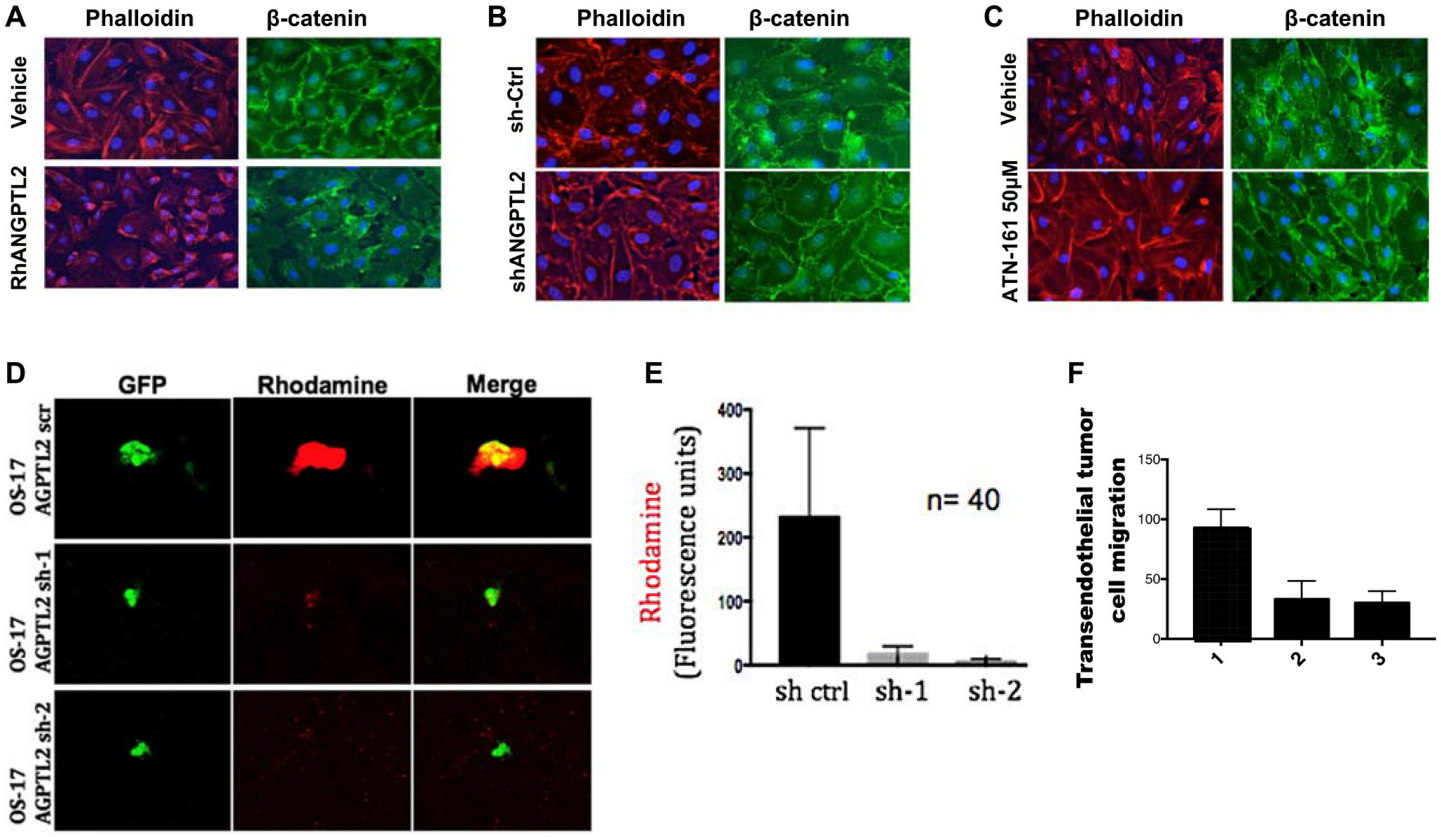
Tumor-secreted ANGPTL2 promotes osteosarcoma metastasis by mediating endothelial monolayer disruption, lung capillary permeability, and trans-endothelial tumor cell migration. (**A**) HUVEC monolayers were grown to confluence on fibronectin-coated slides and then treated with either vehicle (PBS) or human recombinant ANGPTL2 protein (rhANGPTL2) for 24h. Slides were subsequently fluorescently stained with anti-phalloidin, and anti-β-catenin antibody. (**B**) HUVEC monolayers were treated for 24 h with media conditioned by control OS17 osteosarcoma cells that express ANGPTL2 (ctrl) or knockdown OS17-shANGPTL2 (sh) cells. Samples were stained for β-catenin and phalloidin. (**C**) Administration of ANGPTL2 receptor inhibitor, ATN-161, prevents disruption of vascular endothelial cell-cell junctions. HUVEC monolayers were treated for 24 h with media conditioned by OS-17 cells in the presence of drug. Subsequently, samples were stained for β-catenin and phalloidin. ((A–B) Quantitative analysis of paracellular gap formation is shown in Supplementary Figure 7) (**D**) GFP-labeled indicated OS17 cells were injected via the tail vein and allowed to lodge in the lungs. One day post injection, a rhodamine-dextran dye was injected into circulation. Three hours after dye injection, lungs were extracted and frozen sections were obtained. Representative confocal images are shown here of cells with and without accumulation of dye in the lung parenchyma. (**E**) Images were obtained as described in (D) with control or ANGPTL2 knockdown OS17 cells. A region of interest was drawn around the GFP-labeled cells and the amount of dextran dye was quantified based on rhodamine emissions. *n* = 40 cells; error bars indicate s. e. m; *p*-values calculated using the one-tailed unpaired *t*-test. (**F**) OS17-shCtr (1), OS17-sh1ANGPTL2 (2) or OS17-sh2ANGPTL2 (3) were seeded into trans-well inserts that were previously covered with a HUVEC monolayer. Cells that migrated cross the endothelial layer into the bottom side of the transwell membrane were quantified with Volocity software. *n* = 15, error bars indicate s. e. m; *p*-values calculated using the one-tailed unpaired *t*-test.

After a successful establishment of pre metastatic nice formation, circulating cancer cells need to disrupt tight endothelial junctions within the lung to extravasate and seed lung tissues. Interestingly, previous studies showed that the induction of angiopoetin like-4 (ANGPTL4), a member of angiopoietins, increased extravasation of breast tumor cells in the lung. Consequently, we investigated whether ANGPTL2 affects endothelial cells in a manner that would cause loss of tight junctions and passage of tumor cells across the endothelial barrier. Human vascular endothelial cells (HUVEC) were grown to confluence. These monolayers were then exposed to media containing recombinant human ANGPTL2, control media (Figure 4A), or media conditioned by either control OS17 osteosarcoma cells or by ANGPTL2-depleted OS17 cells (Figure 4B). In each case, ANGPTL2 caused acute disruption of endothelial cell-cell junctions. Staining for β-catenin (an adherens junction component) or for phalloidin (an component of the actin cytoskeleton), suggested an ANGPTL2-dependent loss of monolayer integrity (Figure 4A and 4B). We additionally evaluated the ability of ATN-161 (a selective small peptide antagonist of integrin α5β1) to rescue the disruption of endothelial junctions. As shown in Figure 4C, HUVEC monolayers that were exposed to media conditioned by OS17 cells showed significant disruption of endothelial junctions. However, administration of ATN-161 in the conditioned media clearly rescued the disruption of endothelial junctions (Figure 4C) suggesting that targeting ANGPTL2 receptor by ATN-161 might prevent emergence of pulmonary metastasis and prolong overall survival in osteosarcoma models as explored below. To demonstrate vessel permeability *in vivo*, GFP-labeled OS17 cells either expressing shRNA targeting ANGPTL2 or scramble (control) were inoculated into SCID mice. One day post-tumor cell inoculation, animals were injected with a rhodamine-conjugated dextran. The lungs were then collected and retained rhodamine quantitated and evaluated using fluorescent microscopy. The rhodamine signal was essentially undetectable in the lungs of mice that not inoculated with cancer cells (data not shown). In animals that received tail vein inoculation of tumor cells, however, diffuse areas of rhodamine signal surrounded cancer cells that arrested within the lung (Figure 4D). Lungs from mice inoculated with OS17 cells overexpressing ANGPTL2 showed approximately 5-fold higher rhodamine signal, as determined by fluorescent area (Figure 4E). Lastly, to test the functional effect of ANGPTL2 on cell migration across an endothelial layer, endothelial monolayers were grown on transwell tissue culture inserts. OS17 cells overexpressing ANGPTL2 passed through these layers into the lower chamber of the transwell with twice the efficience of that seen with OS17-shANGPTL2 cells (Figure 4F). Collectively, our data suggest that ANGPTL2 alters vascular endothelial cell-cell adhesion to permit extravasaion of osteosarcoma cells in the lung.

### ΔNp63 drives ANGPTL2 expression through interacting NF-κB binding site

**Figure 5:**
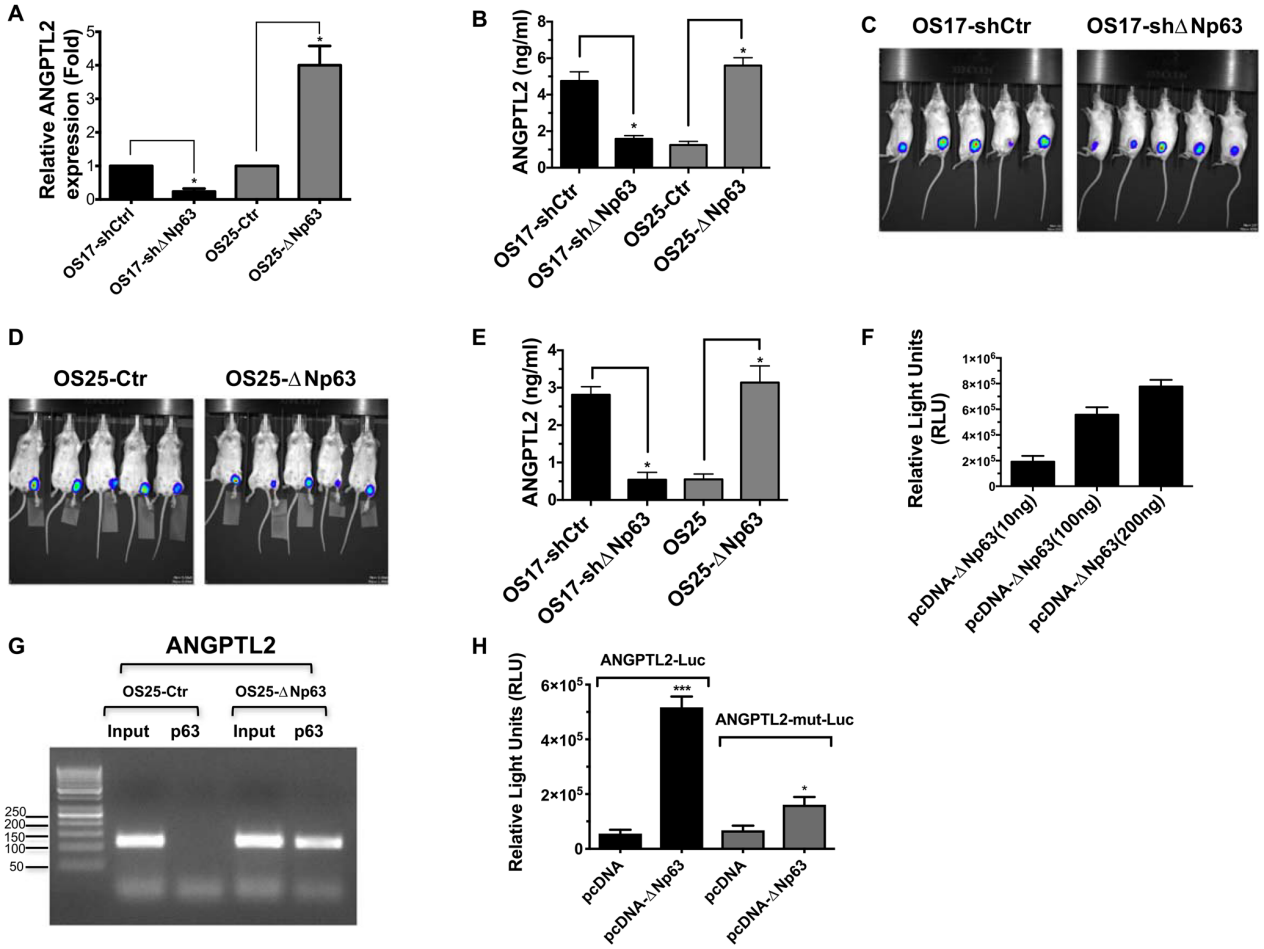
ΔNp63 induces ANGPTL2 expression and secretion. (**A**) qRT-PCR was used to assess ANGPTL2 mRNA levels in indicated osteosarcoma cells. (**B**) Conditioned medium from indicated cells were collected and used for human ANGPTL2 ELISA (Takara). In all experiments, ^*^ *P* values < 0.05 were considered significant. (**C–D**) Indicated luciferase expressing osteosarcoma cells were injected into the tibia of SCID mice. A representative bioluminescence image showing inoculated tumor cells after 7 days. (**E**) Serum from tumor-bearing animals was obtained post 7 days of tumor inoculaton. Subsequently, serum levels of ANGPTL2 were determined using a human ANGPTL2 ELISA kit (Takara). (**F**) ANGPTL2 promoter luciferase construct co-transfected with increasing amounts of ΔNp63 plasmid into HEK-293 cells. Luciferase assay performed in triplicate wells (mean ± S. D) 48 h after transfection as described in materials and methods. (**G**) ChIP assay demonstrate binding of ΔNp63 on ANGPTL2 promoter containing NF-κB site. Immunoprecipitated samples from indicated cell lines were analyzed by PCR using the primers as described in methods. (**H**) Induction of ANGPTL2 luciferase reporter activity by ΔNp63 expression in 293T cells correlates well with the presence of NF-κB response elements. ANGPTL2mutNF-κB luciferase reporter construct was generated as described in materials and methods and assayed similarly. Unpaired Student’s *t*-tests, ^*^ *p* < 0.05, ^***^ *p* < 0.001 vs control.

We next examined the underlying molecular mechanism of high levels of ANGPTL2 secretion in osteosarcoma. We and others demostrated that osteosarcoma xenografts, cell lines and patient samples express high-levels of ΔNp63 [23–25]. To interrogate the role of ΔNp63 in ANGPTL2 regulation in osteosarcoma, we first utilized our OS17-shΔNp63 and OS25-ΔNp63 cell lines expressing high or low basal levels of this gene [26]. As shown in Figure 5A–5B, both mRNA and secretion level of ANGPTL2 in osteosarcoma cells depend on ΔNp63 expression. Subsequently, we wondered whether ΔNp63 expressing primary tumors in the bone milieu would indeed secrete detectable ANGPTL2 levels in an *in vivo* orthotopic mouse models. Therefore, luciferase-expressing osteosarcoma cells were injected into the tibia of SCID mice (Figure 5C–5D) and serum samples were isolated from blood of tumor bearing animals after 4 weeks. As shown in Figure 5E, we found that ANGPTL2 levels in serum is significantly correlated with ΔNp63 expression in tibial tumors. Furthermore, to validate our findings, we examined whether ΔNp63 can activate the ANGPTL2 promoter *in vitro*. To this end, we cloned the human ANGPTL2 promoter (1716bp) into a luciferase reporter plasmid. As shown in Figure 5F, ΔNp63 enhances the ANGPTL2 promoter activity in a dose-dependent manner. The binding of several transcription factors to the ANGPTL2 promoter has previously been determined experimentally [27]. While ΔNp63 lacks a transcriptional activation domain, it retains DNA binding activity. One could postulate that ΔNp63 recruits another transcription factor to the ANGPTL2 promoter, thereby inducing its expression. For example, our previous study demonstrated that ΔNp63, RelA, and cRel members of the NF-κB family can interact together to affect transcription of NF-κB/Rel target genes [28]. To test whether ΔNp63 smiliarly binds ANGPTL2 promoter region, which contains known NF-κB/Rel regulatory element, we first conducted ChIP assay with primer pairs covering NF-κB binding site on ANGPTL2 promoter. As shown in Figure 5H, binding of ΔNp63 to NF-κB site on ANGPTL2 promoter suggests direct regulation of ANGPTL2 by ΔNp63. To support this finding further, we co-transfected ΔNp63 with ANGPTL2 reporter construct either containing wild type or mutated NF-κB-binding site. As shown in Figure 5G, ΔNp63 was able to increase transactivation of the wild type ANGPTL2 promoter, however only a minimal increase in transactivation of the NF-κB mutated promoter was observed. Together, these results demonstrated that ΔNp63 drives ANGPTL2 expression via NF-κB binding site in osteosarcoma.

### Targeting ANGPTL2 receptor signaling prevents formation of lung metastasis in preclinical studies

**Figure 6:**
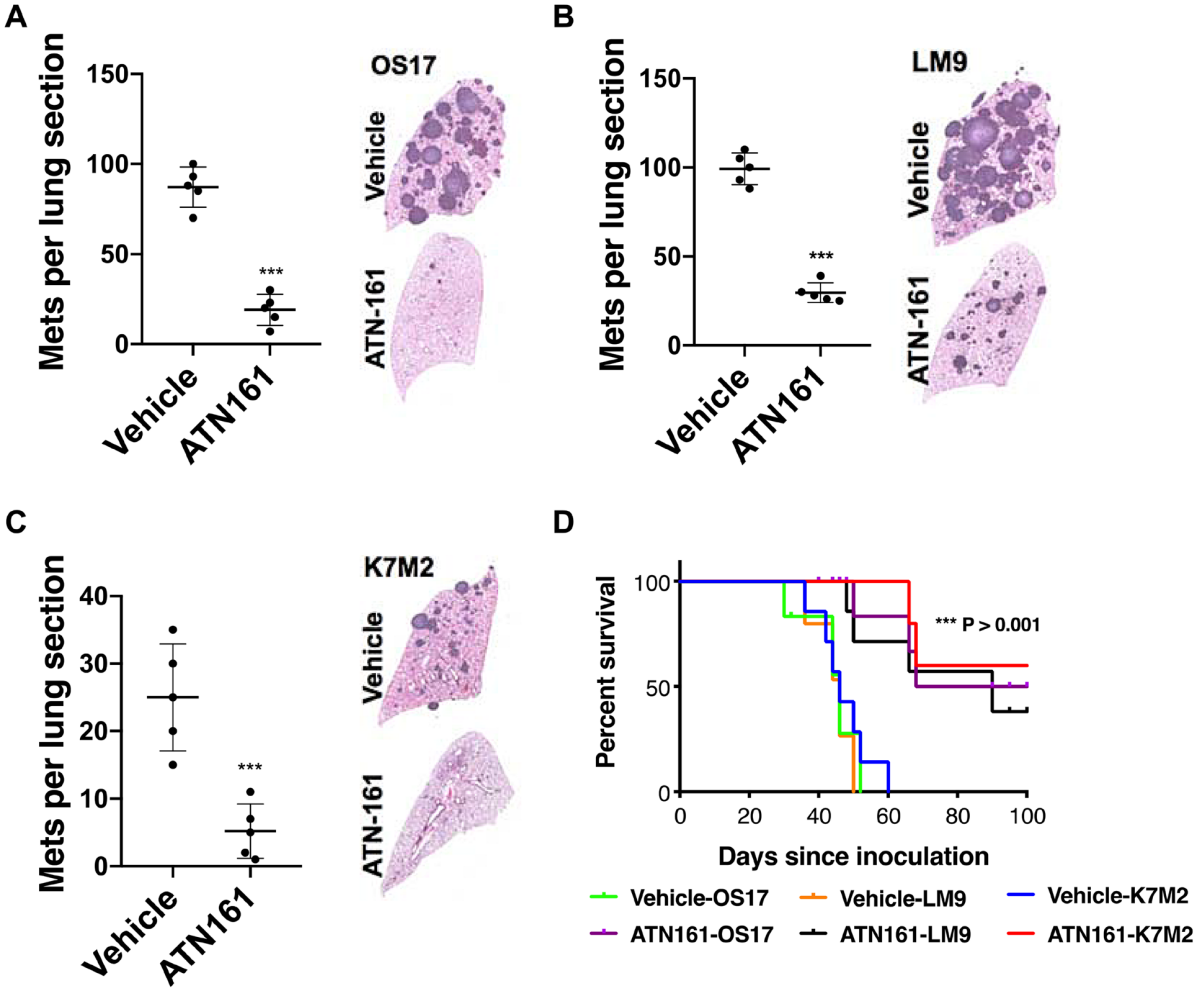
ATN-161 treatment significantly lower metastatic burden and increased overall survival. (**A–C**) SCID mice were inoculated with OS-17 cells via tail vein (A), whereas K7M2 (B) and LM9 (C) cells were inoculated in immunocompetent BALB/c mice. Animals were treated with vehicle alone (PBS) or ATN-161 (1 mg/kg thrice a week) for 6 weeks (*n* = 5, per group). A representative lung section from each group is shown. The number of lung sections with metastatic nodules were compared with the Mann–Whitney *U* test. ^***^ *p* < 0.001. (**D**) Kaplan–Meier survival analysis of the mice shown. Treatment of ATN-161 was continued post injection until day 42, and stopped. Mice that received ATN-161 (1 mg/kg three times a week) experienced significantly better outcomes than vehicle treated group (*n* = 10 mice per group). Mice surviving beyond 100 days were euthanized.

We lastly wondered whether pharmacologic intervention of ANGPTL2 receptor (α5β1) has an effect on metastatic burden in an *in vivo* metastasis mouse model. We utilized the non–RGD-based integrin binding peptide (ATN-161) that has advanced to clinical trials (clinicaltrials. gov). ATN-161 is well tolerated, and has been investigated in a phase 1 trials alone or in combination with chemotherapy in adult patients with solid tumors [29]. As shown in Figure 6A–6C, animals treated with ATN-161 developed significantly less numbers of metastatic foci compared to vehicle treated animals. Importantly, our survival studies demonstrated that targeting ANGPTL2 receptor by ATN-161 prolonged overall survival in osteosarcoma models Figure 6D, thereby laying the groundwork for future clinical trials in children affected with this devastating disease.

## DISCUSSION

ANGPTL2 is a mediator of chronic inflammation and inflammatory carcinogenesis. Here, we report that ANGPTL2 secretion is elevated in osteosarcoma patients when compared to healthy donors. The biological and clinical significance of ANGPTL2 remains unknown in human cancer. Nevertheless, recent studies have shown ANGPTL2 as a potential serum biomarker for colorectal [30], gastric [31] and esophageal cancer [32]. While the genetic alterations that drive osteosarcomagenesis have become progressively well-characterized in the last 5 years, no molecular marker has thus far proved to have more significance or value in predicting response to treatment than the clinical markers already in use [33]. Therefore, novel biomarkers are needed to stratify patients and to enhance existing techniques to interrogate treatment response, for outcome to improve. Future studies will reveal whether ANGPTL2 in serum acts as a biomarker for osteosarcoma progression.

The pre-metastatic niche represents an abnormal, microenvironment that while devoid of cancer cells favors tumor growth. Little is known about the mechanisms involved in this process and how these impact host cells in pre-metastatic organs. The tumor-derived signals that initiate innate immune responses in specific tissues remains a critical but poorly defined process. Here, we investigated whether lung epithelial cells activated by tumor secreted ANGPTL2 might be essential for primary tumor-induced neutrophil recruitment in lung and for subsequent pre-metastatic niche formation in osteosarcoma. Our study strongly suggests that tumor-secreted ANGPTL2 initiates programs that lead to neutrophil recruitment within the lung, a process essential for the lung colonization in spontaneous models of osteosarcoma pulmonary metastasis. In addition to the contribution of tumor-secreted factors to pre-metastatic niche formation, other tumor-independent processes may contribute to dissemination and colonization. Processes related to surgery and wound healing, infection and aging alter the local inflammatory milieu and may help to create a microenvironment that is permissive or that actively promotes colonization by circulating tumor cells [34, 35]. Together, our results should be interpreted with caution.

Lung vascular endothelial junctions form barriers that prevent the extravasation of blood cells. In addition its role in pre metastatic nice formation, we determined that ANGPTL2 drives a loosening of vascular endothelial tight junctions and adherens junctions, creating openings within the capillary wal that permit the passage of osteosarcoma cells. These observations suggest a primary role for ANGPTL2 in osteosarcoma cell extravasation, consistent with the known role of the angiopoietin and angiopoietin-like factors in vascular remodeling [36–39]. We further determined that ΔNp63, an oncogenic isoform of p63, drives ANGPTL2 gene expression and its subsequent secretion from the osteosarcoma cells. We identified that NF-κB binding element on ANGPTL2 promoter is crucial for ΔNp63-mediated transcriptional activity. Furthermore, we explored the therapeutic effects of ANGPTL2 receptor inhibition by integrin binding peptide (ATN-161) in preclinical studies. We revealed that ATN-161 treatment significantly inhibited lung metastasis and prolonged survival suggesting possible therapeutic utility of ATN-161 in early stage osteosarcoma in children.

The mechanisms by which primary tumors initiate pre-metastatic niche formation (PMN) remain poorly understood. In this study, we investigated whether lung epithelial cells might be essential for primary tumor-induced neutrophil recruitment to lung and the role that these infiltrating cells play in osteosarcoma PMN formation using spontaneous metastatic models. We demonstrated that ANGPTL2 played a vital role in the progression of osteosarcoma metastasis by inducing pre-metastatic lung formation and extravasation. Furthermore, our preclinical data suggested that ANGPTL2 signaling might be a potential target for the treatment of osteosarcoma metastasis. Together, results generated from our studies will have an impact not only in osteosarcoma, but other tumors in which ANGPTL2 signaling is known to be deregulated.

## MATERIALS AND METHODS

### Patient samples and cell culture

Blood was obtained from 18 osteosarcoma patients newly diagnosed or patients with osteosarcoma currently receiving treatment at Nationwide Children’s Hospital. Control blood was obtained from ten healthy patients. BD Vacutainer^®^ EDTA blood collection tubes were used for the blood collection. Breifly, blood samples were transferred into 15 ml conical tubes and centrifuged at 800 g for 7 minutes at 4°C. Subsequently, ANGPTL2 serum levels were analysed by ELISA (Takara). Written informed consent for the use of blood samples was obtained from all patients and the protocol for the present study was approved by the Institutional Review Boards of the Nationwide Children’s Hospital. OS17 was derived from an early passage of the OS17 patient-derived xenograft [40]. OS25 and OHS cells were a gift from Oystein Fodstad’s lab at the Radium Hospital in Oslo. OS17-shΔNp63 and OS25-ΔNp63 were described in [26]. All cells were grown in RPMI supplemented with 10% FBS. K7M2 cells were obtained from ATCC (CRL-2836) and cultured as suggested. LM9 cells are a gift from Hideki Yoshikawa’s lab at Osaka Medical Center for Cancer and Cardiovascular Diseases in Osaka, Japan. Murine osteosarcoma K7M2 and LM9 cells were cultured in DMEM supplemented with 10% FBS.

### Mice, tumor models and preclinical studies

Conditional knockout Itga5 (integrin5α) mouse were obtained from Taconic. Sftpc-CreER^T2^ mice were obtained from the Jackson Laboratory. For the intratibial injections, 5 × 10^5^ osteosarcoma cells were injected into the tibia of 6–8 weeks old mice as described in [41, 42]. After primary tumors reached around 800 mm^3^ (range from 4 to 5 weeks), primary tumor containing leg was amputated as described in [41]. Eight weeks after amputation, mice were euthanized; lungs were harvested, insufflated, fixed, sectioned, and stained. Number of metastases per section was quantified. 10 mice each for the experimental and control groups were used. For the preclinical studies, 6 to 8 weeks old female CB17-SCID mice (that retain normal NK and myeloid cell function) were inoculated via tail vein with 1 × 10^6^ OS cells (day 0). Mice received ATN-161 (1 mg/kg I. V., 3 times/week for 6 consecutive weeks), or vehicle. For K7M2 and LM9 cells, immunocompetent BALB/c mice were used, and treatments were performed similarly as in *scid* mice. Mice were monitored twice a week by measuring body weight and eBCS score as described in [43]. Mice demonstrating greater than 10% body weight loss or eBCS less than 8 were euthanized by the carbon dioxide method and tissues harvested, lungs insufflated, and processed as above. Mice not demonstrating metastatic disease burden (presumably dying from other causes) were censored in the survival analysis. For the Kaplan–Meier survival analysis, 10 mice each for the experimental and control groups were used. Mice were housed in a group of up to 5 per cage in special SPF cages which included autoclaved bedding material. All mice were bred and housed at the Nationwide Children’s Hospital in a specific-pathogen free facility. All animal study procedures were carried out in accordance with National Institutes of Health guide for the care and use of Laboratory animals and conducted with approval of the Nationwide Children’s Hospital Institutional Animal Care and Use Committee.

### Lung tissue dissociation, cell isolation and flow cytometry analysis

Lung tissue dissociation, cell isolation and flow cytometry experiments were performed as described in [18]. Briefly, single-cell suspensions from lungs were stained with following antibodies, and T cells (CD45^+^ CD3^+^), B cells (CD45^+^ CD19^+^), macrophages (CD45^+^ CD11b+Gr1^–^), dendritic cells (CD45^+^ CD11c^+^ MHCII^+^), neutrophils (CD45^+^ CD11b^+^Gr1^+^), epithelial cells (CD45^-^ Epcam^+^), and AT-II cells (CD45^-^ SftpD^+^). All flow cytometry analysis was performed with BD-LSR II (BD Biosciences) and data were analyzed with FlowJo software.

### Lentiviral production, quantitative reverse transcription PCR (RT-qPCR), Western blot and ELISA

Specific oligonucleotides targeting ANGPTL2 (sh1: CCTGAGAGCGAGTATTATAAG, sh2: TGGCACAACGGCAAGCAGTTC) and sh control (CCTAAGGTTAAGTCGCCCTCG) were designed and cloned into pLKO.1 cloning vector according to the protocol recommended by Addgene (Cambridge, MA, USA). Subsequently, lentivirus production and infections were performed as described in Addgene’s pLKO.1 protocol. RNA isolation, reverse transcription and western blotting were perfermed similar in [23, 44]. β-Actin antibody is from Santa Cruz and integrin-α5 antibody is from Abcam. Bv8 (Prok2), CXCL1, CXCL2, CXCL5, CXCL12 and GAPGH TaqMan^®^ Gene Expression Assays were purchased from Applied Biosystems. ELISA kits for mouse CXCL1, CXCL2, CXCL5 and CXCL12 were purchased from R&D Systems and assays were performed according to the manufacturer's instructions.

### Immunofluorescence and *in vivo* lung permeability assays

Immunofluorescence and *in vivo* lung permeability assays were performed as described in [39]. Briefly, tumor cells were infected by GFP expressing lentivirus (Cellomics Technology, Hallethorpe, MD, USA) and inoculated into the lateral tail vein. One day post inoculation, mice were injected intravenously with rhodamine-conjugated dextran (70 kDa, Invitrogen) at 2 mg per 20 g body weight. After 3 h, mice were sacrificed; lungs were extracted and lung sections were examined by confocal microscopy for vascular leakage. A region of interest (ROI) was drawn around the GFP-labeled cells and the amount of dextran dye was quantified based on rhodamine emissions. *n* = 40 cells; error bars indicate s. e. m; *p*-values calculated using the one-tailed unpaired *t*-test.

### Chromatin immunoprecipitation (ChIP) and luciferase assay

ChIP assay was performed as described in [45] with p63 antibody (4A4, Santa Cruz Biotechnology, Santa Cruz, CA, USA). The ΔNp63-associated chromatin was analyzed from OS25-ΔNp63 cells via quantitative real time PCR (qRT-PCR) using primer sets flanking the NF-κB binding site (Forward, ACTCAGGTGTGAAGTCACAGAG, Reverse, GCATCGGAGCTGCTCGAAGTTA). Indicated plasmids were transfected along with corresponding luciferase reporter vector into 293T cells. The human ANGPTL2 promoter (1719bp) was cloned from human genomic DNA (BioChain institute Inc., Newark, CA, USA) by PCR (Forward- ATAGGTACCTTGCTCACGTGTCTGCGGCTG and Reverse- GATCTCGAGCCGCCAGAGGAAACTGTG) and subcloned into the pGL3 (Promega, Madison, WI, USA) luciferase reporter vector. The ANGPTL2mutNF-κB luciferase reporter constructs were generated from the pGL3-ANGPTL2-Luc2 construct with the QuikChange™ II Site-Directed Mutagenesis Kit (Agilent, Santa Clara, CA, USA). All luciferase assays were performed according to the manufacturer's instructions (Promega, Madison, WI, USA).

### Statistical analysis

Data were graphed and analyzed using Graphpad Prism 7 (GraphPad Software). Error bars represent mean ± SD from triplicate measurements from one experiment. One representative experiment is showing. Differences between two groups were analyzed by unpaired Student’s *t*-test. The numbers of lung sections with metastatic nodules were compared either with ordinary Two-way Anova analysis or the Mann–Whitney *U* test as described in figure legends. Survival curves were plotted using the Kaplan–Meier method, and log-rank tests were used to compare curves between groups.

## SUPPLEMENTARY MATERIALS



## References

[1] Peinado H , Zhang H , Matei IR , Costa-Silva B , Hoshino A , Rodrigues G , Psaila B , Kaplan RN , Bromberg JF , Kang Y , Bissell MJ , Cox TR , Giaccia AJ , et al. Pre-metastatic niches: organ-specific homes for metastases. Nat Rev Cancer. 2017; 17:302–317. 10.1038/nrc.2017.6. 28303905

[2] Kaplan RN , Riba RD , Zacharoulis S , Bramley AH , Vincent L , Costa C , MacDonald DD , Jin DK , Shido K , Kerns SA , Zhu Z , Hicklin D , Wu Y , et al. VEGFR1-positive haematopoietic bone marrow progenitors initiate the pre-metastatic niche. Nature. 2005; 438:820–827. 10.1038/nature04186. 16341007 PMC2945882

[3] Sceneay J , Smyth MJ , Möller A . The pre-metastatic niche: finding common ground. Cancer Metastasis Rev. 2013; 32:449–464. 10.1007/s10555-013-9420-1. 23636348

[4] Wagner WR , Griffith BP . Reconstructing the lung. Science. 2010; 329:520–522. 10.1126/science.1194087. 20671176

[5] Whitsett JA , Alenghat T . Respiratory epithelial cells orchestrate pulmonary innate immunity. Nat Immunol. 2015; 16:27–35. 10.1038/ni.3045. 25521682 PMC4318521

[6] Hartl D , Latzin P , Hordijk P , Marcos V , Rudolph C , Woischnik M , Krauss-Etschmann S , Koller B , Reinhardt D , Roscher AA , Roos D , Griese M . Cleavage of CXCR1 on neutrophils disables bacterial killing in cystic fibrosis lung disease. Nat Med. 2007; 13:1423–1430. 10.1038/nm1690. 18059279

[7] Juncadella IJ , Kadl A , Sharma AK , Shim YM , Hochreiter-Hufford A , Borish L , Ravichandran KS . Apoptotic cell clearance by bronchial epithelial cells critically influences airway inflammation. Nature. 2013; 493:547–551. 10.1038/nature11714. 23235830 PMC3662023

[8] Brody JS , Steiling K . Interaction of cigarette exposure and airway epithelial cell gene expression. Annu Rev Physiol. 2011; 73:437–456. 10.1146/annurev-physiol-012110-142219. 21090967

[9] Takahashi H , Ogata H , Nishigaki R , Broide DH , Karin M . Tobacco smoke promotes lung tumorigenesis by triggering IKKbeta- and JNK1-dependent inflammation. Cancer Cell. 2010; 17:89–97. 10.1016/j.ccr.2009.12.008. 20129250 PMC2818776

[10] Fridlender ZG , Sun J , Kim S , Kapoor V , Cheng G , Ling L , Worthen GS , Albelda SM . Polarization of tumor-associated neutrophil phenotype by TGF-beta: “N1” versus “N2” TAN. Cancer Cell. 2009; 16:183–194. 10.1016/j.ccr.2009.06.017. 19732719 PMC2754404

[11] Liu Y , Cao X . Immunosuppressive cells in tumor immune escape and metastasis. J Mol Med (Berl). 2016; 94:509–522. 10.1007/s00109-015-1376-x. 26689709

[12] Granot Z , Henke E , Comen EA , King TA , Norton L , Benezra R . Tumor entrained neutrophils inhibit seeding in the premetastatic lung. Cancer Cell. 2011; 20:300–314. 10.1016/j.ccr.2011.08.012. 21907922 PMC3172582

[13] Coffelt SB , Kersten K , Doornebal CW , Weiden J , Vrijland K , Hau CS , Verstegen NJM , Ciampricotti M , Hawinkels L , Jonkers J , de Visser KE . IL-17-producing γδ T cells and neutrophils conspire to promote breast cancer metastasis. Nature. 2015; 522:345–348. 10.1038/nature14282. 25822788 PMC4475637

[14] Cools-Lartigue J , Spicer J , McDonald B , Gowing S , Chow S , Giannias B , Bourdeau F , Kubes P , Ferri L . Neutrophil extracellular traps sequester circulating tumor cells and promote metastasis. J Clin Invest. 2013; 123:3446–3458. 10.1172/JCI67484. 23863628 PMC3726160

[15] Wu CF , Andzinski L , Kasnitz N , Kroger A , Klawonn F , Lienenklaus S , Weiss S , Jablonska J . The lack of type I interferon induces neutrophil-mediated pre-metastatic niche formation in the mouse lung. Int J Cancer. 2015; 137:837–847. 10.1002/ijc.29444. 25604426

[16] Wculek SK , Malanchi I . Neutrophils support lung colonization of metastasis-initiating breast cancer cells. Nature. 2015; 528:413–417. 10.1038/nature16140. 26649828 PMC4700594

[17] Bald T , Quast T , Landsberg J , Rogava M , Glodde N , Lopez-Ramos D , Kohlmeyer J , Riesenberg S , van den Boorn-Konijnenberg D , Hömig-Hölzel C , Reuten R , Schadow B , Weighardt H , et al. Ultraviolet-radiation-induced inflammation promotes angiotropism and metastasis in melanoma. Nature. 2014; 507:109–113. 10.1038/nature13111. 24572365

[18] Liu Y , Gu Y , Han Y , Zhang Q , Jiang Z , Zhang X , Huang B , Xu X , Zheng J , Cao X . Tumor Exosomal RNAs Promote Lung Pre-metastatic Niche Formation by Activating Alveolar Epithelial TLR3 to Recruit Neutrophils. Cancer Cell. 2016; 30:243–256. 10.1016/j.ccell.2016.06.021. 27505671

[19] Kadomatsu T , Endo M , Miyata K , Oike Y . Diverse roles of ANGPTL2 in physiology and pathophysiology. Trends Endocrinol Metab. 2014; 25:245–254. 10.1016/j.tem.2014.03.012. 24746520

[20] Odagiri H , Kadomatsu T , Endo M , Masuda T , Morioka MS , Fukuhara S , Miyamoto T , Kobayashi E , Miyata K , Aoi J , Horiguchi H , Nishimura N , Terada K , et al. The secreted protein ANGPTL2 promotes metastasis of osteosarcoma cells through integrin α5β1, p38 MAPK, and matrix metalloproteinases. Sci Signal. 2014; 7:ra7. 10.1126/scisignal.2004612. 24448647

[21] Lin C , Song H , Huang C , Yao E , Gacayan R , Xu SM , Chuang PT . Alveolar type II cells possess the capability of initiating lung tumor development. PLoS One. 2012; 7:e53817. 10.1371/journal.pone.0053817. 23285300 PMC3527621

[22] Kowanetz M , Wu X , Lee J , Tan M , Hagenbeek T , Qu X , Yu L , Ross J , Korsisaari N , Cao T , Bou-Reslan H , Kallop D , Weimer R , et al. Granulocyte-colony stimulating factor promotes lung metastasis through mobilization of Ly6G+Ly6C+ granulocytes. Proc Natl Acad Sci U S A. 2010; 107:21248–21255. 10.1073/pnas.1015855107. 21081700 PMC3003076

[23] Bid HK , Roberts RD , Cam M , Audino A , Kurmasheva RT , Lin J , Houghton PJ , Cam H . ΔNp63 promotes pediatric neuroblastoma and osteosarcoma by regulating tumor angiogenesis. Cancer Res. 2014; 74:320–329. 10.1158/0008-5472.CAN-13-0894. 24154873 PMC3950294

[24] Cam M , Gardner HL , Roberts RD , Fenger JM , Guttridge DC , London CA , Cam H . ΔNp63 mediates cellular survival and metastasis in canine osteosarcoma. Oncotarget. 2016; 7:48533–48546. 10.18632/oncotarget.10406. 27391430 PMC5217036

[25] Ram Kumar RM , Betz MM , Robl B , Born W , Fuchs B . ΔNp63α enhances the oncogenic phenotype of osteosarcoma cells by inducing the expression of GLI2. BMC Cancer. 2014; 14:559. 10.1186/1471-2407-14-559. 25085524 PMC4125704

[26] Gross AC , Cam H , Phelps DA , Saraf AJ , Bid HK , Cam M , London CA , Winget SA , Arnold MA , Brandolini L , Mo X , Hinckley JM , Houghton PJ , et al. IL-6 and CXCL8 mediate osteosarcoma-lung interactions critical to metastasis. JCI Insight. 2018; 3:e99791. 10.1172/jci.insight.99791. 30135299 PMC6141177

[27] Thorin-Trescases N , Thorin E . Angiopoietin-like-2: a multifaceted protein with physiological and pathophysiological properties. Expert Rev Mol Med. 2014; 16:e17. 10.1017/erm.2014.19. 25417860

[28] Yang X , Lu H , Yan B , Romano RA , Bian Y , Friedman J , Duggal P , Allen C , Chuang R , Ehsanian R , Si H , Sinha S , Van Waes C , et al. ΔNp63 versatilely regulates a Broad NF-κB gene program and promotes squamous epithelial proliferation, migration, and inflammation. Cancer Res. 2011; 71:3688–3700. 10.1158/0008-5472.CAN-10-3445. 21576089 PMC3443863

[29] Cianfrocca ME , Kimmel KA , Gallo J , Cardoso T , Brown MM , Hudes G , Lewis N , Weiner L , Lam GN , Brown SC , Shaw DE , Mazar AP , Cohen RB . Phase 1 trial of the antiangiogenic peptide ATN-161 (Ac-PHSCN-NH(2)), a beta integrin antagonist, in patients with solid tumours. Br J Cancer. 2006; 94:1621–1626. 10.1038/sj.bjc.6603171. 16705310 PMC2361324

[30] Yoshinaga T , Shigemitsu T , Nishimata H , Kitazono M , Hori E , Tomiyoshi A , Takei T , Yoshida M . Angiopoietin-like protein 2 as a potential biomarker for colorectal cancer. Mol Clin Oncol. 2015; 3:1080–1084. 10.3892/mco.2015.577. 26623054 PMC4534869

[31] Yoshinaga T , Shigemitsu T , Nishimata H , Takei T , Yoshida M . Angiopoietin-like protein 2 is a potential biomarker for gastric cancer. Mol Med Rep. 2015; 11:2653–2658. 10.3892/mmr.2014.3040. 25484242

[32] Ide S , Toiyama Y , Shimura T , Kawamura M , Yasuda H , Saigusa S , Ohi M , Tanaka K , Mohri Y , Kusunoki M . Angiopoietin-Like Protein 2 Acts as a Novel Biomarker for Diagnosis and Prognosis in Patients with Esophageal Cancer. Ann Surg Oncol. 2015; 22:2585–2592. 10.1245/s10434-014-4315-0. 25564164

[33] Kong C , Hansen MF . Biomarkers in Osteosarcoma. Expert Opin Med Diagn. 2009; 3:13–23. 10.1517/17530050802608496. 20574545 PMC2889491

[34] Shibue T , Weinberg RA . Metastatic colonization: settlement, adaptation and propagation of tumor cells in a foreign tissue environment. Semin Cancer Biol. 2011; 21:99–106. 10.1016/j.semcancer.2010.12.003. 21145969

[35] Weilbaecher KN , Guise TA , McCauley LK . Cancer to bone: a fatal attraction. Nat Rev Cancer. 2011; 11:411–425. 10.1038/nrc3055. 21593787 PMC3666847

[36] Camenisch G , Pisabarro MT , Sherman D , Kowalski J , Nagel M , Hass P , Xie MH , Gurney A , Bodary S , Liang XH , Clark K , Beresini M , Ferrara N , et al. ANGPTL3 stimulates endothelial cell adhesion and migration via integrin αvβ3 and induces blood vessel formation *in vivo* . J Biol Chem. 2002; 277:17281–17290. 10.1074/jbc.M109768200. 11877390

[37] Gale NW , Thurston G , Hackett SF , Renard R , Wang Q , McClain J , Martin C , Witte C , Witte MH , Jackson D , Suri C , Campochiaro PA , Wiegand SJ , et al. Angiopoietin-2 is required for postnatal angiogenesis and lymphatic patterning, and only the latter role is rescued by Angiopoietin-1. Dev Cell. 2002; 3:411–423. 10.1016/S1534-5807(02)00217-4. 12361603

[38] Parikh SM , Mammoto T , Schultz A , Yuan HT , Christiani D , Karumanchi SA , Sukhatme VP . Excess circulating angiopoietin-2 may contribute to pulmonary vascular leak in sepsis in humans. PLoS Med. 2006; 3:e46. 10.1371/journal.pmed.0030046. 16417407 PMC1334221

[39] Padua D , Zhang XH , Wang Q , Nadal C , Gerald WL , Gomis RR , Massagué J . TGFβ primes breast tumors for lung metastasis seeding through angiopoietin-like 4. Cell. 2008; 133:66–77. 10.1016/j.cell.2008.01.046. 18394990 PMC2390892

[40] Houghton PJ , Morton CL , Tucker C , Payne D , Favours E , Cole C , Gorlick R , Kolb EA , Zhang W , Lock R , Carol H , Tajbakhsh M , Reynolds CP , et al. The pediatric preclinical testing program: description of models and early testing results. Pediatr Blood Cancer. 2007; 49:928–940. 10.1002/pbc.21078. 17066459

[41] Chaffee BK , Allen MJ . A clinically relevant mouse model of canine osteosarcoma with spontaneous metastasis. *In Vivo*. 2013; 27:599–603. 23988893 PMC4001249

[42] Charan M , Dravid P , Cam M , Audino A , Gross AC , Arnold MA , Roberts RD , Cripe TP , Pertsemlidis A , Houghton PJ , Cam H . GD2-directed CAR-T cells in combination with HGF-targeted neutralizing antibody (AMG102) prevent primary tumor growth and metastasis in Ewing sarcoma. Int J Cancer. 2019 10 17. 10.1002/ijc.32743. [Epub ahead of print]. 31621900 PMC7440656

[43] Khanna C , Prehn J , Yeung C , Caylor J , Tsokos M , Helman L . An orthotopic model of murine osteosarcoma with clonally related variants differing in pulmonary metastatic potential. Clin Exp Metastasis. 2000; 18:261–271. 10.1023/A:1006767007547. 11315100

[44] Cam M , Bid HK , Xiao L , Zambetti GP , Houghton PJ , Cam H . p53/TAp63 and AKT regulate mammalian target of rapamycin complex 1 (mTORC1) signaling through two independent parallel pathways in the presence of DNA damage. J Biol Chem. 2014; 289:4083–4094. 10.1074/jbc.M113.530303. 24366874 PMC3924274

[45] Cam M , Charan M , Welker AM , Dravid P , Studebaker AW , Leonard JR , Pierson CR , Nakano I , Beattie CE , Hwang EI , Kambhampati M , Nazarian J , Finlay JL , et al. ΔNp73/ETS2 complex drives glioblastoma pathogenesis- targeting downstream mediators by rebastinib prolongs survival in preclinical models of glioblastoma. Neuro Oncol. 2019 11 25. 10.1093/neuonc/noz190. [Epub ahead of print]. 31763674 PMC7058445

