# Supplementary material for: Tumor secreted ANGPTL2 facilitates recruitment of neutrophils to the lung to promote lung pre-metastatic niche formation and targeting ANGPTL2 signaling affects metastatic disease
Source: Oncotarget. 2020 Feb 4;11(5):510–22. doi: 10.18632/oncotarget.27433 (PMC7007290; doi:10.18632/oncotarget.27433)
Supplement: Supplementary file 1 [file oncotarget-11-510-s001.pdf]

Tumor secreted ANGPTL2 facilitates recruitment of neutrophils to the lung to promote lung pre-metastatic niche formation and targeting ANGPTL2 signaling affects metastatic disease

SUPPLEMENTARY MATERIALS

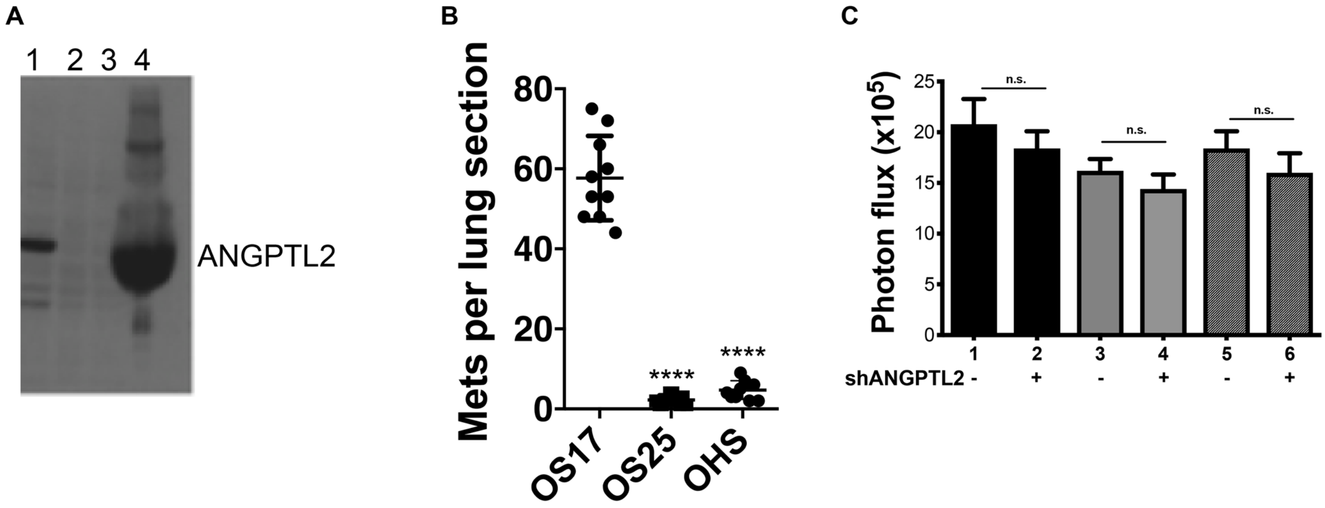

Supplementary Figure 1: Expression of ANGPTL2 correlates with lung-colonization efficiency. (**A**) Immunoblot analysis of ANGPTL2 protein expression in osteosarcoma cell lines. Cell extracts were analyzed by western blot with antibodies as shown. Lanes: 1. OS17, 2. OS25, 3. OHS, 4. Supernatant from OS17. (**B**) Mice were injected with 1×10^6 osteosarcoma cells via tail vein and were euthanized 42 days after tail vein injections. Quantification reveals significantly higher number of metastases (mets) in the OS-17 sections relative to both OS-25 and OHS (*for each group 10 mice were used*). $^{****}P < 0.0001$ relative to OS-17, 1-way Anova with Tukey's post hoc test. (**C**) No significant reduction of the primary tumor growth following ANGPTL2 inhibition. Luciferase expressing 5×10^5 osteosarcoma cells were injected into tibia and all mice were observed for 4 weeks (*for each group 5 mice were used*). Subsequently, the bioluminescence intensity was quantified using Living Image Software. Signal intensity was quantified as the sum of detected photons per second within the region of interest. Graph shows the quantitation of bioluminescence between the groups (n.s. non significant). Lanes: 1. LM9-shCtr, 2. LM9-shANGPTL2, 3. K7M2-shCtr 4. K7M2-shANGPTL2, 5-OS17-shCtr, 6-OS17-shANGPTL2.

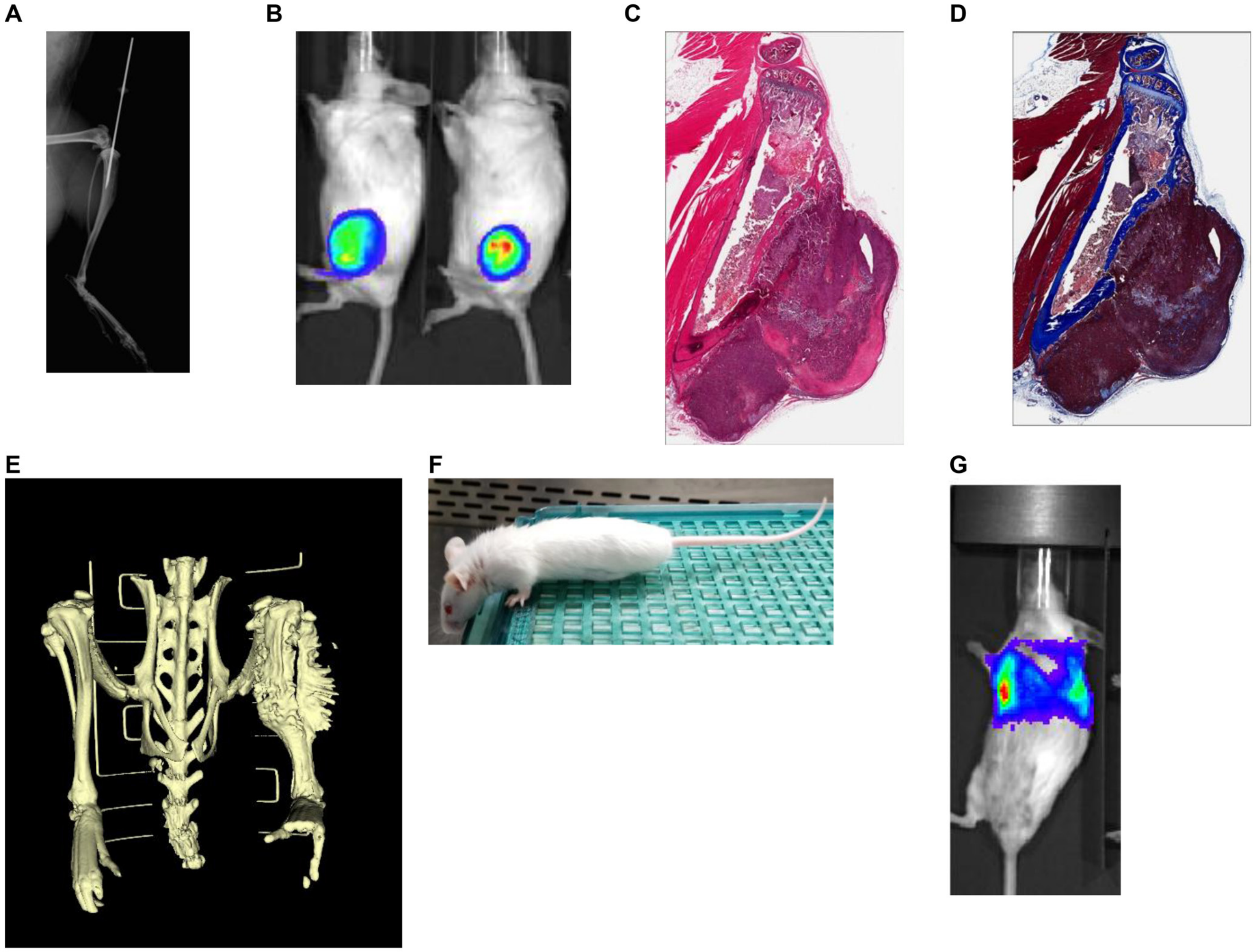

Supplementary Figure 2: Orthotopic mouse models for the studies have been successfully established in our lab. (**A**) Representative X-ray images of a mouse tibia showing the injection procedure. (**B**) Representative image showing intratibial implantation of luciferase labeled OS17 cells in SCID mice. Bioluminescent images were taken four weeks after tibial implantation. (**C–D**) Harvested tibias in H&E and Trichrome staining. Orthotopic tumor showing its extension from the bone to the surrounding soft tissue. (**E**) Representative MicroCT scans taken from the tibia-injected mice (B) showing osteolytic lesion (**F**). Four weeks following intratibial tumor implantation, the tumor-bearing leg was amputated. Picture shows recovered mouse two weeks after amputation. (**G**) A representative image showing lung metastasis eight weeks after amputation.

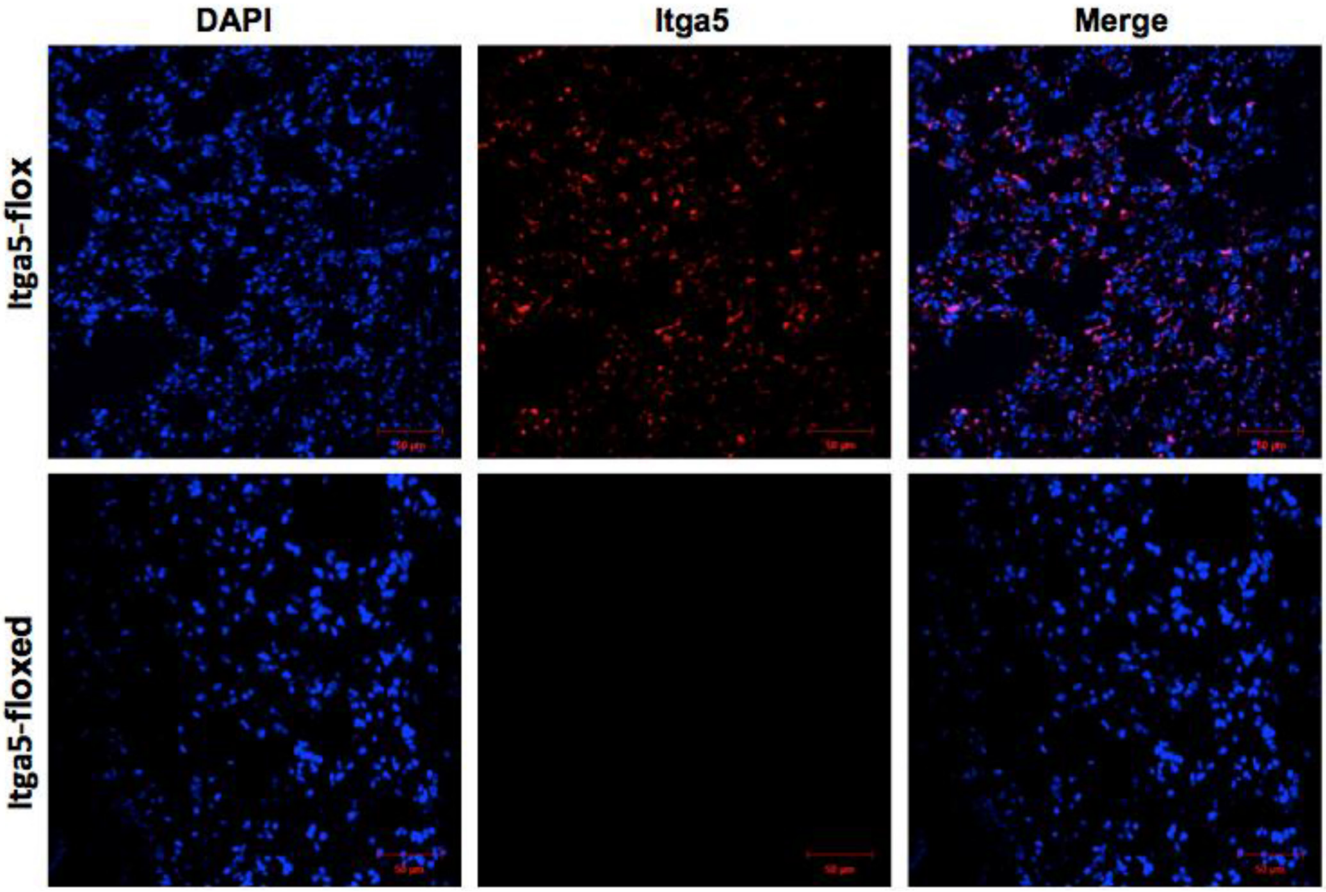

Supplementary Figure 3: Lung sections were immunostained with Itga5 alpha specific antibody and analyzed by immunofluorescence. DAPI was used to counterstain nuclei. Scale bar, 50 µm.

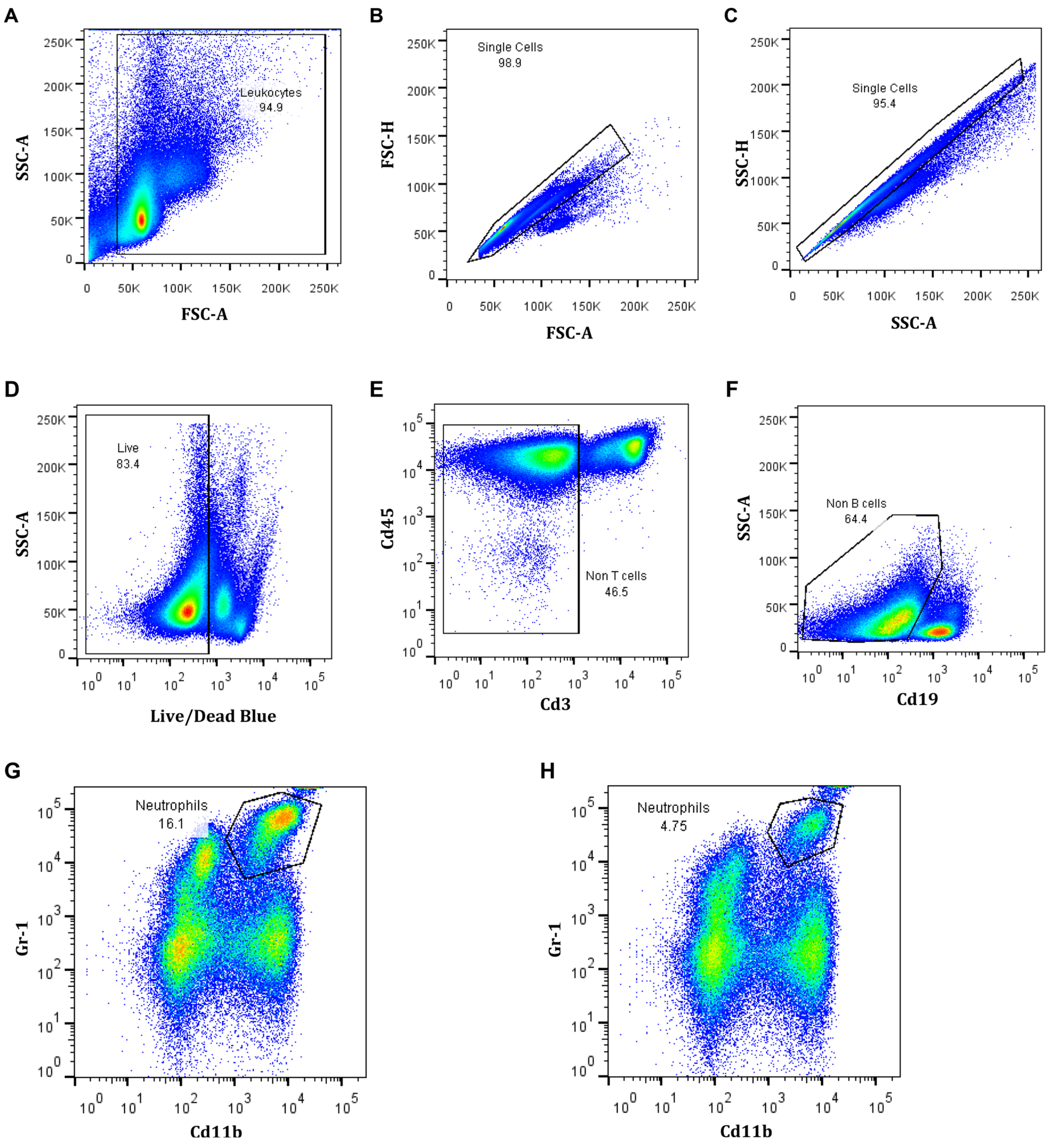

Supplementary Figure 4: A representative flow cytometry gating strategy showing for the neutrophils isolated from lung tissues after two weeks of tumor cell inoculation. (**A**) Scatter gate to exclude debris, (**B–C**) singlets gates to exclude doublets, (**D**) live gate to exclude DAPI+ dead cells, (**E**) gate on CD45+ CD3- leukocytes excluding T cells, (**F**) gate excluding Cd19+ B cells (**G**) gate for neutrophils (CD11b^{+}Gr1^{+}) from WT mice inoculated with K7M2 cells (**H**) CD11b^{+}Gr1^{+} neutrophils from Itga5-floxed mice inoculated with K7M2 cells (gating strategy same as A–F).

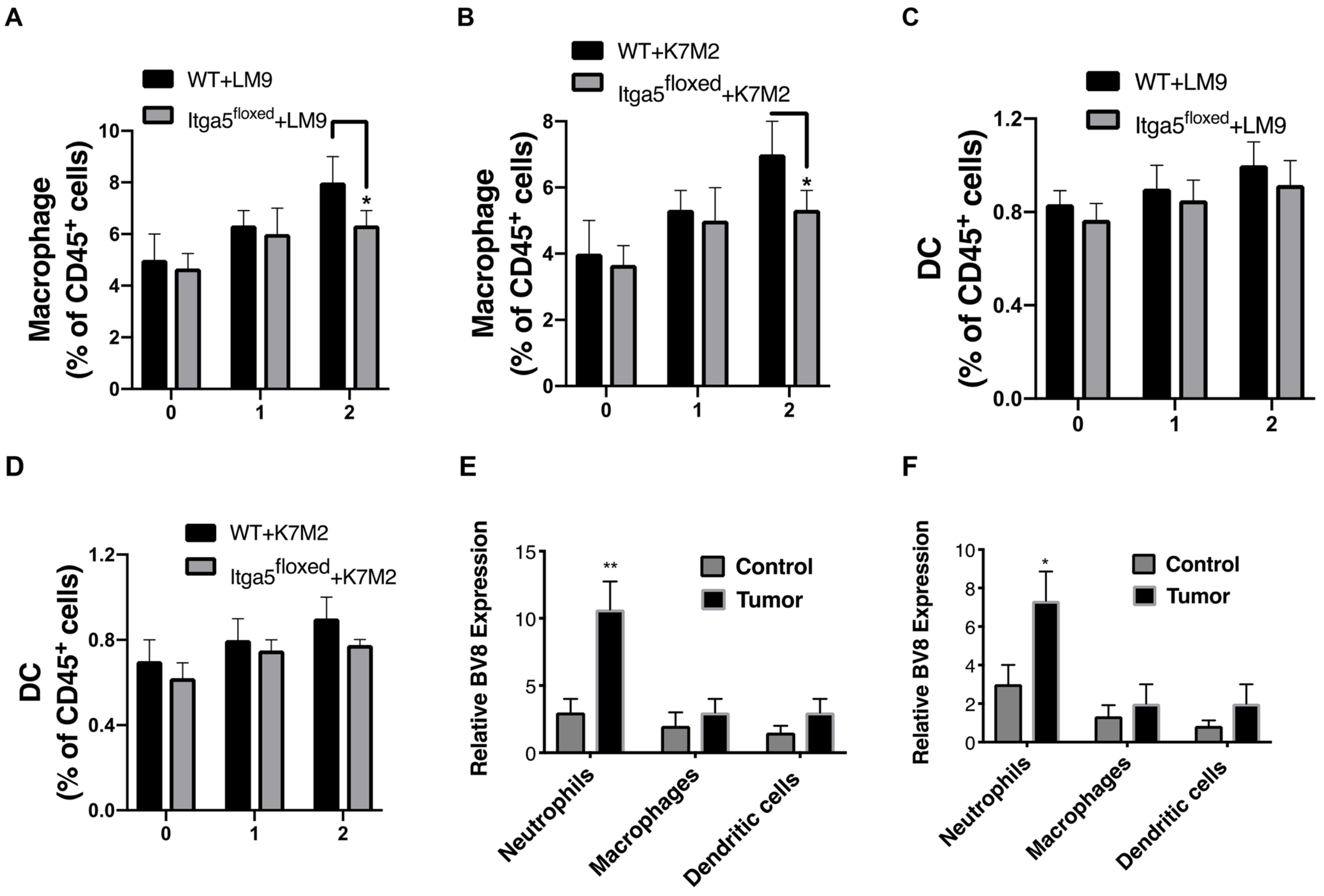

Supplementary Figure 5: Integrin α5β1 receptor deficiency impairs myeloid cell recruitment into the lungs upon tumor inoculation. In contrast to CD45+ CD11b+ $Gr1^+$ neutrophils (Figure 3A–3B), CD11b+$Gr1^-$ macrophages showed a moderate accumulation in lung, whereas $CD45^+$ $CD11c^+$ $MHCII^+$ dendritic cells showed no difference. The proportions of macrophages (**A–B**) or dendritic cells (DCs) (**C–D**) in lung were detected by flow cytometry at 0–1–2 weeks after LM9 or K7M2 tumor inoculation. Unpaired Student's *t*-tests, $^*p < 0.05$. (**E–F**) Gene expression analysis of BV8 in neutrophils, macrophages or dendritic cells derived from the lungs inoculated either with saline (control) or LM9 (E) or K7M2 (F) cells were quantified by qRT-PCR. Results were normalized to GAPDH.

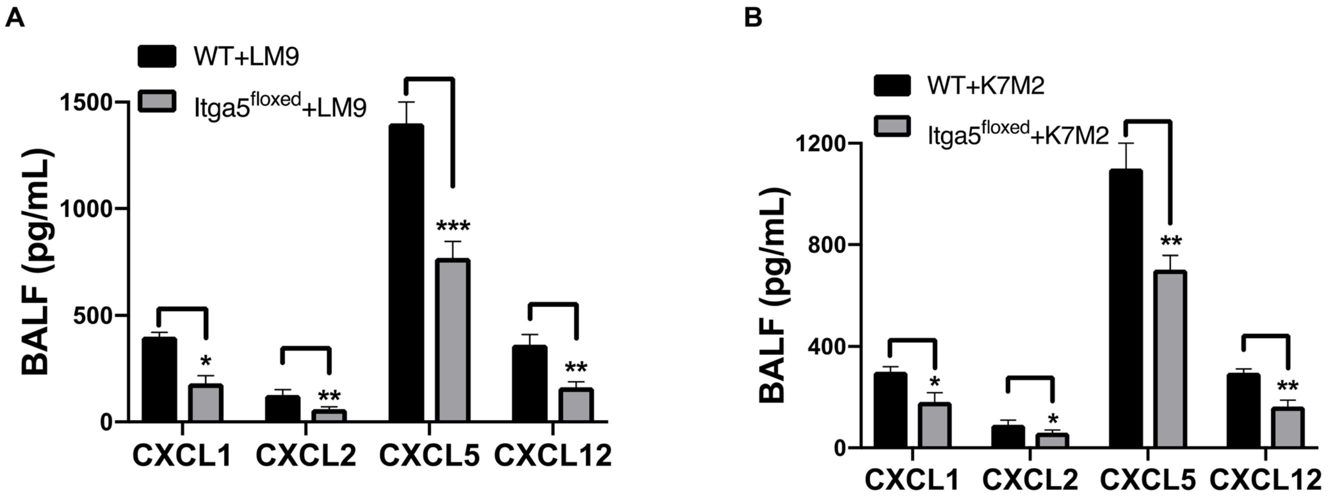

Supplementary Figure 6: Chemokines (CXCL1, CXCL2, CXCL5, and CXCL12) that are known to be required for neutrophil chemotaxis in bronchoalveolar lavage fluid (BALF) are significantly lower in Itg5a$^{-/-}$ mice. BALF was performed through a 20-gauge angiocath ligated into the trachea. A 1.0-ml aliquot of PBS was instilled into mouse lungs and then slowly aspirated 3 times. A 200 µl aliquot of the BAL fluid was placed in a cytospin and centrifuged at 100 *g* for 5 minutes. The supernatant was used to measure indicated chemokine levels by ELISA (R&D Systems). After a week of intratibial injection, BALF were isolated as described above from LM9 (**A**) or K7M2 (**B**) tumor bearing animals. Unpaired Student's *t*-tests, $^{*}p < 0.05$, $^{**}p < 0.01$, $^{***}p < 0.001$.

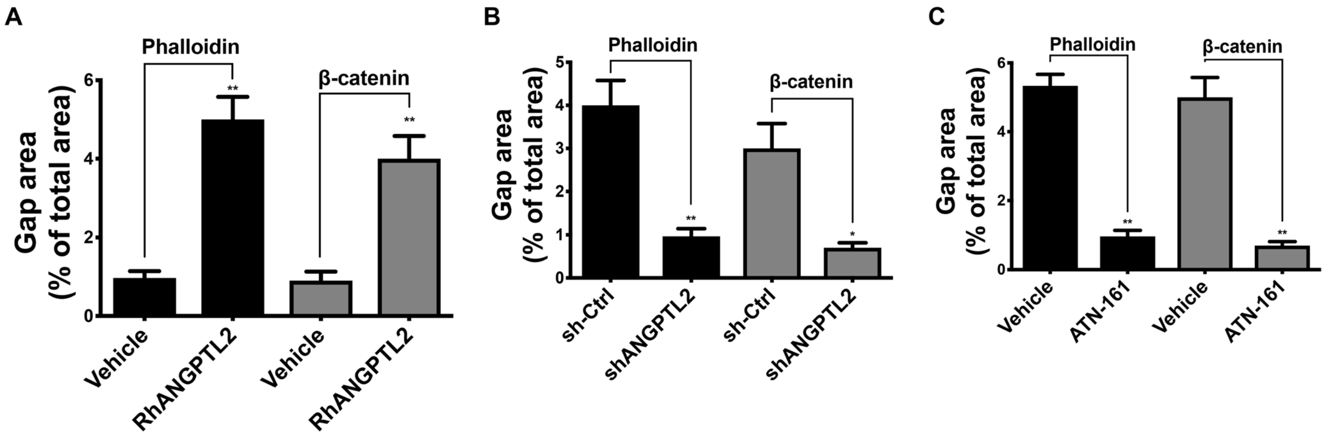

Supplementary Figure 7: ANGPTL2 promotes endothelial monolayer disruption. (**A–C**) The localization of junction proteins and the cell gap were recorded with a laser scanning confocal microscope (Zeiss). Images (n = 3, from each condition) were differentially segmented between gaps and cells based on image gray scale levels. Quantitative analysis of gap formation was performed using Image J software. Data sets were analyzed with unpaired student's *t*-test and significance between two groups is shown $^{*}p < 0.05$, $^{**}p < 0.01$.
